# Targeted Therapy With Immunoconjugates for Multiple Myeloma

**DOI:** 10.3389/fimmu.2020.01155

**Published:** 2020-06-19

**Authors:** Wassilis S. C. Bruins, Sonja Zweegman, Tuna Mutis, Niels W. C. J. van de Donk

**Affiliations:** Department of Hematology, Cancer Center Amsterdam, Amsterdam UMC, Vrije Universiteit Amsterdam, Amsterdam, Netherlands

**Keywords:** multiple myeloma, immunoconjugates, antibody-drug conjugates, immunotoxins, immunocytokines, radioimmunoconjugates, monoclonal antibodies, immunotherapy

## Abstract

The introduction of proteasome inhibitors (PI) and immunomodulatory drugs (IMiD) has markedly increased the survival of multiple myeloma (MM) patients. Also, the unconjugated monoclonal antibodies (mAb) daratumumab (anti-CD38) and elotuzumab (anti-SLAMF7) have revolutionized MM treatment given their clinical efficacy and safety, illustrating the potential of targeted immunotherapy as a powerful treatment strategy for MM. Nonetheless, most patients eventually develop PI-, IMiD-, and mAb-refractory disease because of the selection of resistant MM clones, which associates with a poor prognosis. Accordingly, these patients remain in urgent need of new therapies with novel mechanisms of action. In this respect, mAbs or mAb fragments can also be utilized as carriers of potent effector moieties to specifically target surface antigens on cells of interest. Such immunoconjugates have the potential to exert anti-MM activity in heavily pretreated patients due to their distinct and pleiotropic mechanisms of action. In addition, the fusion of highly cytotoxic compounds to mAbs decreases the off-target toxicity, thereby improving the therapeutic window. According to the effector moiety, immunoconjugates are classified into antibody-drug conjugates, immunotoxins, immunocytokines, or radioimmunoconjugates. This review will focus on the mechanisms of action, safety and efficacy of several promising immunoconjugates that are under investigation in preclinical and/or clinical MM studies. We will also include a discussion on combination therapy with immunoconjugates, resistance mechanisms, and future developments.

## Introduction

Multiple myeloma (MM) is a malignancy of plasma cells, which typically proliferate within the bone marrow. MM is the second most prevalent hematologic malignancy (1), accounting for 159 985 incident cases and 106 105 mortality cases globally in 2018 (2). Over the past decades, the prognosis of MM patients has markedly improved due to the introduction of high-dose melphalan therapy with autologous stem cell transplantation (ASCT), proteasome inhibitors (PI), and immunomodulatory drugs (IMiD). However, the majority of patients eventually become refractory to all available therapies because of the selection of drug-resistant MM clones during treatment (3–5). Also, the outcome remains especially poor for patients with an unfavorable cytogenetic profile (6). Accordingly, further improvements in treatment outcome will require the development of new anti-MM therapies with novel mechanisms of action.

Recently, advances in MM research have led to the emergence of promising treatment approaches such as cellular- and monoclonal antibody (mAb)-based immunotherapies. In this respect, the unconjugated mAbs daratumumab (anti-CD38) and elotuzumab (anti-SLAMF7) have shown impressive anti-MM activity and a beneficial toxicity profile. Although these antibodies have transformed the treatment of both heavily pretreated and newly diagnosed MM, most patients eventually develop resistance to mAbs during the treatment course, which is associated with poor survival (7). Prolonged remission of these patients may eventually be realized by exploring the ways in which these mAb-based therapies can be enhanced.

**Figure 1:**
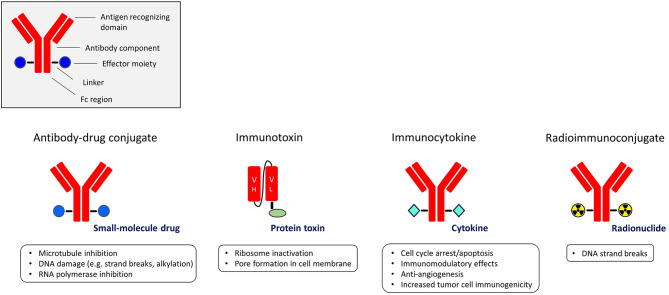
The different subgroups of immunoconjugates with the main functions of their effector moieties.

A highly appealing strategy to exploit the targeting power of antibodies or antibody fragments is to utilize them as carriers of potent effector moieties to the tumor cells. Such immunoconjugates aim to specifically expose target cells to high amounts of cytotoxic or immune-modulating molecules, compared with non-target cells, resulting in an improved therapeutic window. Depending on the effector moiety used, immunoconjugates are classified into four subgroups: antibody-drug conjugates (ADC), immunotoxins, immunocytokines, and radioimmunoconjugates (Figure 1).

In this review, we will focus on the most promising immunoconjugates for the treatment of MM, in different phases of preclinical and clinical development. We will provide an overview of the different classes of immunoconjugates, with a strong focus on mechanisms of action, efficacy, and safety profile. In addition, we will discuss combination strategies with immunoconjugates, off-target toxicities, resistance mechanisms, and future developments.

## Antibody-Drug Conjugates

**Figure 2:**
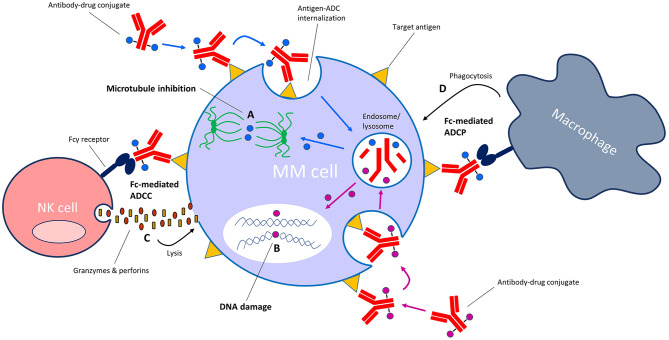
Mechanism of action of antibody-drug conjugates (ADC) with microtubule-inhibiting or DNA-damaging payloads. Following binding of the ADC to its specific target on the cell surface, the antigen-ADC complex is internalized into the cell and the cytotoxic payload is released from the endosome/lysosome into the cytosol. Depending on the type of payload, the payload then either **(A)** inhibits microtubule formation or **(B)** induces damage to cellular DNA (e.g., strand breaks, alkylation). ADCs with an intact Fc tail may also induce Fc-mediated effector functions such as **(C)** antibody-dependent cellular cytotoxicity (ADCC) and/or **(D)** antibody-dependent cellular phagocytosis (ADCP).

ADCs are a rapidly growing class of immunotherapeutic drugs used for the treatment of solid and hematologic malignancies (8–11). An ADC consists of a mAb or mAb fragment which is armed with a cytotoxic drug (also known as the payload, warhead or small-molecule) via a cleavable or non-cleavable linker. When the antibody component binds to its specific cell surface antigen, the antigen-ADC complex is internalized into the cell and the payload is released into the cytoplasm via linker cleavage or linker and/or antibody degradation in the endolysosomes. The payload then interferes with vital cellular functions, resulting in cell death (Figure 2). Thus, ADCs are “guided missiles” that combine the lethal properties of cytotoxic payloads with the selectivity of mAbs. Additionally, some ADCs induce Fc-mediated effector functions including antibody-dependent cellular cytotoxicity (ADCC) and antibody-dependent cellular phagocytosis (ADCP) (12, 13).

The design of ADCs is generally more complex as opposed to unconjugated mAbs—the performance of each ADC component is important for specific anti-tumor activity and safety. Preferably, the antibody targets a tumor-associated antigen displaying a high density on cancer cells as opposed to normal cells, whereas linker stability is critical when the ADC is *en route* toward its target in the bloodstream to avoid ADC disintegration. Suitable payloads are highly cytotoxic at low concentrations, easily conjugatable to antibodies, and stable when administered *in vivo*. Commonly used payloads include anti-mitotic microtubule blockers such as maytansinoid and auristatin, DNA-damaging agents such as pyrrolobenzodiazepine and calicheamicin, and the RNA polymerase inhibitor amanitin (14).

Although the basic idea behind ADCs was already introduced back in the 1960s (15), their success in oncology has especially accelerated in recent years. To date, four ADCs have been granted (conditional) marketing authorization by the Food and Drug Administration (FDA) and European Medicines Agency (EMA) for the treatment of hematologic malignancies: gemtuzumab-ozogamicin (anti-CD33; Mylotarg) for acute myeloid leukemia, brentuximab vedotin (anti-CD30; Adcetris) for Hodgkin lymphoma, inotuzumab ozogamicin (anti-CD22; Besponsa) for acute lymphoid leukemia, and polatuzumab vedotin (anti-CD79b; Polivy) for diffuse large B-cell lymphoma. For MM, several ADCs have reached the clinical phase of drug testing, while others are still in preclinical stages (Table 1). In the next section, we will discuss several of these ADCs with a focus on ADCs that are most advanced in clinical evaluation, or ADCs that have shown notable preclinical efficacy.

**Table 1:** List of antibody-drug conjugates investigated for multiple myeloma.

**Drug name**	**Target**	**Effector moiety**	**Development state**	**Clinical trial**	**Treatment regimen**	**Status**	**References**
Belantamab mafodotin (GSK2857916)	BCMA (CD269)	Monomethyl auristatin F	Clinical	NCT02064387 (DREAMM-1); phase 1	Bela mono	Completed	(13, 16–21)
				NCT03525678 (DREAMM-2); phase 2	Bela mono	Active, not recruiting	
				NCT03715478 (DREAMM-3); phase 3	Bela mono vs. pom + dex	Recruiting	
				NCT03848845 (DREAMM-4); phase 1/2	Bela + pembro	Recruiting	
				NCT04126200 (DREAMM-5); phase 1/2	Bela + innovative anti-cancer drugs	Recruiting	
				NCT03544281 (DREAMM-6); phase 1/2	Bela + len + dex or Bela + bort + dex	Recruiting	
				NCT03715478 (ALGONQUIN); phase 1/2	Bela + pom + dex	Recruiting	
				(DREAMM-7); phase 3	Bela + bort + dex vs. dara + bort + dex	Planned	
				(DREAMM-8); phase 3	Bela + pom + dex vs. bort + pom + dex	Planned	
				NCT04091126 (DREAMM-9); phase 3	Bela + bort + len + dex vs. bort + len + dex	Recruiting	
				(DREAMM-10); phase 3	Bela + novel agent vs. SOC	Planned	
MEDI2228	BCMA (CD269)	Pyrrolo-benzodiazepine	Clinical	NCT03489525; phase 1	MEDI2228 mono	Recruiting	(22–26)
HDP-101	BCMA (CD269)	Amanitin	Preclinical	-	-	-	(27–30)
CC-99712	BCMA (CD269)	Undisclosed	Clinical	NCT04036461; phase 1	CC-99712 mono	Recruiting	-
AMG 224	BCMA (CD269)	Maytansinoid DM1	Clinical	NCT02561962; phase 1	AMG 224 mono	Active, not recruiting	-
SG1-vcMMAF8 SG2-vcMMAF8 SG3-vcMMAF8	BCMA (CD269)	Monomethyl auristatin F	Preclinical	-	-	-	(31)
BCMA-024 BCMA-077	BCMA (CD269)	Duostatin 5.2	Preclinical	-	-	-	(32)
CD38-077	CD38	Duostatin 5.2	Preclinical	-	-	-	(33)
Dara-DM4	CD38	Maytansinoid DM4	Preclinical	-	-	-	(34)
FOR46	CD46	Monomethyl auristatin F	Clinical	NCT03650491; phase 1	FOR46 mono	Recruiting	(35)
SGN-CD48A	CD48	Monomethyl auristatin E	Clinical	NCT03379584; phase 1	SGN-CD48A mono	Terminated (due to overall benefit/risk profile)	(36, 37)
Lorvotuzumab mertansine (IMGN901)	CD56	Maytansinoid DM1	Clinical	NCT00346255; phase 1 NCT00991562; phase 1 NCT02420873; phase 2	Lorvo mono Lorvo + len + dex Lorvo mono	Completed Completed Completed	(12, 38–42)
STRO-001	CD74	DBCO-linker-maytansinoid (SC236)	Clinical	NCT03424603; phase 1	STRO-001 mono	Recruiting	(43, 44)
Milatuzumab-doxorubicin (IMMU-110)	CD74	Doxorubicin	Clinical	NCT01101594; phase 1/2	Mila mono	Completed	(45)
Indatuximab ravtansine (BT062)	CD138	Maytansinoid DM4	Clinical	NCT00723359; phase 1 NCT01001442; phase 1/2a NCT01638936; phase 1/2a	Inda mono single-dose Inda mono multi-dose Inda + len + dex and inda + pom + dex	Completed Completed Completed	(46–50)
B-B4-DM1	CD138	Maytansinoid DM1	Preclinical	-	-	-	(51)
DFRF4539A	FcRL5 (CD307)	Monomethyl auristatin E	Clinical	NCT01432353; phase 1	DFRF4539A mono	Completed	(52, 53)
Anti-FcRL5-SPDB-DM4	FcRL5 (CD307)	Maytansinoid DM4	Preclinical	-	-	-	(52)
Azintuxizumab vedotin (ABBV-838)	SLAMF7 (CD319)	Monomethyl auristatin E	Clinical	NCT02951117; phase 1b NCT02462525; phase 1/1b	Azin + venetoclax + dex Azin & azin + pom + dex	Withdrawn Terminated (No Go decision)	(54–56)
SGN-CD352A	SLAMF6 (CD352)	Pyrrolo-benzodiazepine	Clinical	NCT02954796; phase 1	SGN-CD352A mono	Terminated (sponsor decision)	(57)
MEDI7247	ASCT2 (SLC1A5)	Pyrrolo-benzodiazepine	Clinical	NCT03106428; phase 1	MEDI7247 mono	Active, not recruiting	(58)
M24-DOX	Matriptase	Doxorubicin	Preclinical	-	-	-	(59)

*azin, azintuxizumab vedotin; bela, belantamab mafodotin; bort, bortezomib; dara, daratumumab; dex, dexamethasone; inda, indatuximab ravtansine; len, lenalidomide; lorvo, lorvotuzumab mertansine; mila, milatuzumab-doxorubicin; mono, monotherapy; pembro, pembrolizumab; pom, pomalidomide; SOC, standard-of-care; vs, versus*.

### ADCs Targeting B Cell Maturation Antigen

#### Belantamab Mafodotin (GSK2857916)

Belantamab mafodotin consists of a humanized, afucosylated B cell maturation antigen (BCMA)-specific IgG1 mAb fused to the payload monomethyl auristatin F (MMAF) by a non-cleavable linker (13, 16–20). BCMA is a member of the tumor necrosis factor receptor superfamily that is exclusively expressed on late-stage B cells and plasma cells, as well as on MM cells (16, 60, 61). BCMA promotes the survival of long-lived plasma cells and MM cells following interaction with its ligands A Proliferation Inducing Ligand (APRIL) and B-cell Activating Factor (BAFF) (60–63). Because BCMA expression is restricted to (malignant) plasma cells and a subset of mature B cells, it is an attractive target for anti-MM immunotherapy (64–66).

Belantamab mafodotin showed potent and pleiotropic anti-MM activity in preclinical *in vitro* and *in vivo* models, without significant off-target cytotoxicity on BCMA-negative immune effector cells or bone marrow stromal cells (BMSC) (13, 16). The MMAF payload induces anti-proliferative (cell cycle arrest in G2/M phase) and pro-apoptotic anti-MM effects. In addition, belantamab mafodotin triggers Fc-receptor mediated effector functions including NK cell-mediated ADCC and macrophage-mediated ADCP via its afucosylated Fc tail. Furthermore, belantamab mafodotin induces immunogenic cell death (21), and also inhibits NF-kB signaling by competing with APRIL and BAFF for binding to BCMA (13).

Based on these preclinical findings, the ADC was evaluated in a first-in-human, phase 1 dose-escalation/expansion study (DREAMM-1) (17, 18). Thirty-eight patients were enrolled in the dose-escalation phase. The MTD was not identified, but based on clinical safety and efficacy data, the recommended dose for the expansion phase was defined as 3.4 mg/kg administered every three weeks (17). In the expansion phase, 35 additional patients were included (>4 lines: 57%; PI-refractory: 97%; IMiD-refractory: 94%; daratumumab-refractory: 40%) (18). The most reported adverse events included corneal events (69% of patients, mostly grade 1/2 [54%]), thrombocytopenia (grade 3/4 in 34% of patients), and anemia (grade 3 in 17% of patients). In the expansion phase, at least partial response (PR) was observed in 60% of patients, with 54% achieving a very good partial response (VGPR) or better. Median progression-free survival (PFS) was 12.0 months, with a median duration of response of 14.3 months.

The DREAMM-2 study was initiated to further assess the efficacy and safety of two doses of single-agent belantamab mafodotin (2.5 or 3.4 mg/kg administered every 3 weeks) in patients with ≥ three prior lines of treatment including disease refractory to an IMiD or PI, and disease refractory or intolerant to a CD38-targeting antibody (19). This two-arm phase 2 study enrolled 196 relapsed/refractory MM patients (>4 lines: 83%; bortezomib-refractory: 76%; carfilzomib-refractory: 61%; lenalidomide-refractory: 89%; pomalidomide-refractory: 82%; daratumumab-refractory: 96%). The overall response rate (ORR) was 31% in the 2.5 mg/kg cohort and 34% in the 3.4 mg/kg cohort, with at least VGPR in 19 and 20% of patients treated with 2.5 and 3.4 mg/kg, respectively. The median PFS was 2.9 months in the 2.5 mg/kg cohort and 4.9 months in the 3.4 mg/kg cohort. At the time of analysis, overall survival (OS) data were immature. Toxicity was similar to what was observed in the previous DREAMM-1 study with keratopathy, anemia, and thrombocytopenia as the most reported side effects. Keratopathy (changes to corneal epithelium) was the most common reason for treatment discontinuation, dose reduction, or dose delay. Keratopathy could be effectively managed with dose reductions and delays, and the use of artificial tears. Prophylactic use of corticosteroid eye drops seems to be ineffective. The mechanism of corneal damage is probably related to non-specific uptake of the MMAF payload by actively dividing corneal epithelial cells via macropinocytosis (67). Notably, corneal events were also observed in other clinical trials with other ADCs containing MMAF (68). Infusion-related reactions occurred in 19% of patients (predominantly during the first infusion) and were mostly grade 1/2. The 2.5 mg/kg dose was associated with a more favorable toxicity profile and comparable anti-MM activity when compared to the 3.4 mg/kg dose, and therefore selected as the recommended dose for future studies. The FDA has currently granted a priority review of belantamab mafodotin for the treatment of relapsed/refractory MM patients who have previously received an IMiD, PI and anti-CD38 mAb, whereas the EMA has granted PRIME designation to the ADC.

In preclinical models, preincubation of effector peripheral blood mononuclear cells (PBMC) with the IMiD lenalidomide enhanced belantamab mafodotin-mediated ADCC against MM cell lines or primary MM cells (13). Also, cotreatment of MM cells with both agents in absence of PBMCs enhanced the direct cytotoxicity of the ADC. Furthermore, *in vitro* anti-MM activity was augmented by combining belantamab mafodotin with a gamma-secretase inhibitor, which inhibits BCMA shedding from the cell surface, thereby increasing surface BCMA density and decreasing soluble BCMA levels (20). These preclinical studies provide the rationale for the evaluation of belantamab mafodotin in combination with other agents in planned or already ongoing clinical studies (Table 1).

#### MEDI2228

MEDI2228 is a novel ADC comprised of a fully human BCMA-binding Ab1 antibody conjugated to DNA-damaging agent pyrrolobenzodiazepine via a protease-cleavable linker (22–26). In preclinical models, MEDI2228 effectively killed the majority of MM cell lines and primary MM cells from relapsed/refractory patients, also in the presence of BMSCs or high levels of soluble BCMA. Activity was independent of surface BCMA levels on MM cells, and unaffected by the stimulation of MM cells with IL-6. BCMA-negative cells were not killed. *In vivo*, a single injection of MEDI2228 eliminated tumors in MM mouse models. Importantly, MEDI2228 and bortezomib synergistically decreased viability of drug-sensitive and drug-resistant MM cells *in vitro*, and *in vivo* cotreatment with both agents in mice augmented the eradication of MM tumors and improved survival. In contrast to an MMAF analog, the PBD payload induced DNA damage responses (DDR) and synergized with multiple DDR-inhibitors (23, 25). A phase 1 dose-escalation/-expansion study of MEDI2228 as monotherapy in relapsed/refractory patients is currently ongoing (NCT03489525).

#### HDP-101

HDP-101 consists of a BCMA-specific antibody fused to the RNA polymerase inhibitor amanitin. (27–30). HDP-101 induced dose-dependent lysis of MM cell lines and primary MM cells, even when BCMA expression was low. Notably, the ability of HDP-101 to kill primary MM cells was higher compared with the activity of the same anti-BCMA mAb conjugated to MMAF. BCMA-negative cell lines and patient BMSCs were spared from cytotoxicity. Interestingly, HDP-101 was especially potent in preclinical models with a knockout of tumor suppressor TP53 and knockdown of RNA polymerase POLR2A, which mimics the deletion of 17p in a subtype of high-risk MM patients (29). HDP-101 also had anti-MM activity in mouse models. In a dose-escalation study in cynomolgus monkeys, a drug serum half-life of around 12 days was reached. HDP-101 was well-tolerated with only moderate and transient increases in liver enzymes and lactate dehydrogenase. A first-in-human clinical trial application is expected to be submitted in the second half of 2020 (69).

#### Other BCMA-Targeting ADCs

Table 1 provides an overview of the other BCMA-targeting ADCs AMG-224, CC-99712, and SG1-auristatin.

### ADCs Targeting Other Markers

#### Indatuximab Ravtansine (BT062)

This ADC is comprised of the microtubule-blocker maytansinoid DM4 linked by cleavable disulfide linkers to a chimeric anti-CD138 (syndecan-1) IgG4 antibody (46–50). CD138 is a transmembrane heparan sulfate proteoglycan involved in cell proliferation, cell migration, cell-cell—and cell-matrix adhesion (70, 71). Within the human hematopoietic system, CD138 is considered a specific marker for normal and malignant plasma cells (72, 73). However, CD138 is also expressed on epithelial- and endothelial cells, as well as on fibroblasts and hepatocytes (70, 71, 74).

Indatuximab ravtansine induced dose-dependent cytotoxicity against CD138-positive MM cell lines and primary MM cells, while sparing CD138-negative PBMCs from healthy donors (46). Treatment of MM cell lines revealed G2-M cell cycle arrest with subsequent caspase-dependent apoptosis as a mechanism of action. Cytotoxicity could not be inhibited by addition of exogenous IL-6 or insulin-like growth factor-1, or coculture with BMSCs. Interestingly, indatuximab ravtansine inhibited adhesion of MM cells to BMSCs. Indatuximab ravtansine also inhibited tumor growth and improved survival in MM mouse models.

Altogether, this supported initiation of a phase 1 study (50). Indatuximab ravtansine as a single agent was initially administered once every 3 weeks in 31 relapsed/refractory MM patients (median of prior lines: 7; 100% received prior treatment with bortezomib, 100% lenalidomide or thalidomide, 61% ASCT). The maximum tolerated dose (MTD) was defined as 160 mg/m^2^ with two patients developing dose-limiting toxicities (DLT) at the dose of 200 mg/m^2^ including mucosal inflammation and palmar-plantar erythrodysesthesia syndrome. In this study, the safety profile of indatuximab ravtansine was generally acceptable but the ORR was low (3.2%), and therefore another administration schedule was explored in a phase 1/2a study (50). In this multi-dosing schedule, indatuximab ravtansine was administered on days 1, 8 and 15 of a four-week cycle to 34 relapsed/refractory MM patients (median of prior lines: 5; 100% received prior treatment with bortezomib, 100% lenalidomide or thalidomide, 65% ASCT). Two out of four patients experienced DLTs at 160 mg/m^2^ (one neutropenia, and one transaminitis), and therefore the MTD was established as 140 mg/m^2^. The safety profile was acceptable, with the most frequently reported adverse events being diarrhea (37%), fatigue (34%), anemia (31%), and nausea (23%) (88% of adverse events grade 1/2). At least PR was achieved in 5.9% of patients, minor response (MR) in 8.8%, and stable disease (SD) in 61.8%. Median PFS and median OS were 3.0 and 26.7 months, respectively.

Based on preclinical data showing synergy between indatuximab ravtansine and lenalidomide (47, 49), a second phase 1/2a study (NCT01638936) was initiated in which the ADC (administered on day 1, 8, and 15) was combined with low-dose dexamethasone and either lenalidomide or pomalidomide in a four-week cycle (48). Forty-seven patients with 1-6 prior therapies were treated in combination with lenalidomide/dexamethasone (three patients at 80 mg/m^2^, 38 at 100 mg/m^2^, 6 at 120 mg/m^2^). The recommended phase 2 dose (RP2D) of indatuximab ravtansine in this cohort was identified as 100 mg/m^2^. Again, most frequent toxicities were diarrhea, fatigue, and nausea (approximately 90% were grade 1/2). Of the 43 patients evaluable for response, 33 patients (77%) achieved a PR or better with a median response duration of 21.0 months. In the 13 patients who were previously exposed to both lenalidomide and bortezomib, at least PR was achieved in 54%. For the whole group of patients, the median PFS of indatuximab ravtansine combined with lenalidomide/dexamethasone was 16.4 months. An additional 17 patients, with at least two prior therapies including both lenalidomide and bortezomib, were treated with indatuximab ravtansine at the RP2D in combination with pomalidomide/dexamethasone. Of the 14 patients evaluable for response, 79% achieved a PR or better. The median PFS had not yet been reached after a median follow-up of 7.5 months. However, to the best of our knowledge, the clinical development of this drug has been halted, which may be related to its minimal single agent activity. The limited activity as monotherapy may be explained by the mechanism of action of the maytansinoid payload, which affects only proliferating cells. In addition, several studies show that CD138 can be rapidly shed from the cell surface (75), whereby high levels of soluble CD138 may act as a drug sink.

#### Lorvotuzumab Mertansine (IMGN901)

This ADC consists of the cytotoxic maytansinoid DM1 payload (microtubule inhibitor) attached to the humanized anti-CD56 IgG1 antibody N901 via a disulfide linker (38–42). CD56, originally described as neuronal cell adhesion molecule (NCAM) (76–78), is a cell surface glycoprotein that has an important role in cell adhesion (76, 79, 80). CD56 is expressed on neurons and skeletal muscle cells (77, 81, 82), and within the human hematopoietic system on NK cells, a subset of T cells, monocytes and dendritic cells (83–85). While being absent from normal plasma cells (86), MM cells from the majority of patients express CD56 (38, 87, 88). Notably, CD56-negative MM is associated with reduced bone destruction (89, 90), but increased frequency of extramedullary spread including plasma cell leukemia (91, 92).

In preclinical studies, lorvotuzumab mertansine impaired the survival of CD56-expressing MM cells in a dose-dependent manner, even when adherent to BMSCs (38). CD56-negative tumor cells were unaffected. Cytotoxicity was associated with G2-M cell arrest and apoptosis. In addition, the ADC induced NK cell-mediated ADCC through its Fc tail (12). Cytotoxicity was not correlated with surface CD56 expression (38). Additional *in vitro* testing revealed additive-to-synergistic anti-MM effects when combining lorvotuzumab mertansine with thalidomide, dexamethasone, or melphalan (39). Lorvotuzumab mertansine also suppressed tumor growth in mouse models, and combination treatment with either bortezomib or lenalidomide improved the *in vivo* anti-MM activity of the ADC (40).

The potent preclinical activity formed the rationale for a phase 1 trial in which lorvotuzumab mertansine was evaluated as a single agent in 37 relapsed/refractory patients (>3 prior treatment lines in 78% of patients) with CD56-positive MM (42). Patients received lorvotuzumab mertansine on days one and eight of a three-week cycle. The MTD was defined as 112 mg/m^2^ after two patients experienced a DLT at 140 mg/m^2^ (grade 3 fatigue, grade 3 renal failure). The toxicity profile was acceptable, although 51% of patients experienced treatment-related peripheral neuropathy (5.3% grade 3-4). Among 35 patients evaluable for response, anti-MM activity was modest: 5.7% of patients achieved PR, 11.4% MR, and 42.9% SD. Median PFS was 26.1 weeks.

In a second phase 1 trial with relapsed/refractory MM patients (two median prior lines of treatment; 33% lenalidomide refractory), the combination of lorvotuzumab mertansine (administered on days 1, 8 and 15) with lenalidomide (25 mg on days 1–21) and dexamethasone (40 mg on days 1, 8, 15, and 22) in a four-week cycle resulted in an ORR of 59%, including 31% of patients with a VGPR or better (41). Again, neuropathy (mostly grade 2 or less) was the most common adverse event. Based on the high incidence of neuropathy and limited efficacy, clinical development of lorvotuzumab mertansine for the treatment of MM was discontinued (93).

#### STRO-001

STRO-001 couples the human, aglycosylated CD74-specific IgG1 mAb SP7219 to a cytotoxic maytansinoid payload via a non-cleavable linker (43). CD74, a transmembrane glycoprotein which functions as a receptor for macrophage migration inhibitory factor and as a MHC class II chaperone (94), is expressed in the majority of MM cases (43, 95). Preclinical studies showed that STRO-001 had anti-MM activity against MM cell lines, as well as in mice (43). *In vitro* cytotoxicity tended to positively correlate with CD74 expression levels on MM cell surface, but STRO-001 was also active in cell lines with low CD74 expression. Furthermore, STRO-001 was well-tolerated in cynomolgus monkeys at concentrations ≤ 10 mg/kg (serious DLTs at 30 mg/kg), despite depletion of B cells and monocytes. Preliminary results from a first-in-human phase 1 trial (NCT03424603) with both 11 relapsed/refractory non-Hodgkin lymphoma (NHL) and 14 MM patients showed that STRO-001 monotherapy was safe. The most common toxicity were chills, fatigue, pyrexia, nausea, cough, and infusion reactions (58% of adverse events were grade 1 or 2) (44). At this moment (highest dose tested: 0.91 mg/kg), the MTD has not been reached. One MM patient achieved SD, but up till now no objective responses were observed.

#### FOR46

FOR46 consists of the CD46-specific mAb 23AG2 attached to the effector moiety MMAF via a protease-cleavable linker (35). CD46 is a transmembrane glycoprotein that regulates the activity of the complement system by binding and inactivating C3b and C4b (96). CD46 is overexpressed on MM cells when compared with non-malignant immune cells (35, 97). FOR46 had high and specific cytotoxic activity against MM cell lines and primary MM cells, while non-malignant mononuclear cells were not affected (35). CD46 expression levels correlated positively with the activity of FOR46. Interestingly, because CD46 levels were upregulated on MM cells that were cocultured with BMSCs, FOR46 showed increased killing ability in the presence of BMSCs. Moreover, MM cells from patients with amplification of 1q21 had higher levels of CD46 when compared with samples from patients without this genetic abnormality. This resulted in higher activity of FOR46 in samples from patients with gain of chromosome 1q21, indicating that this ADC may be of particular interest for this high-risk patient group. In mouse models, FOR46 reduced tumor burden in a dose-dependent fashion, and improved survival compared with control mice.

## Immunotoxins

**Figure 3:**
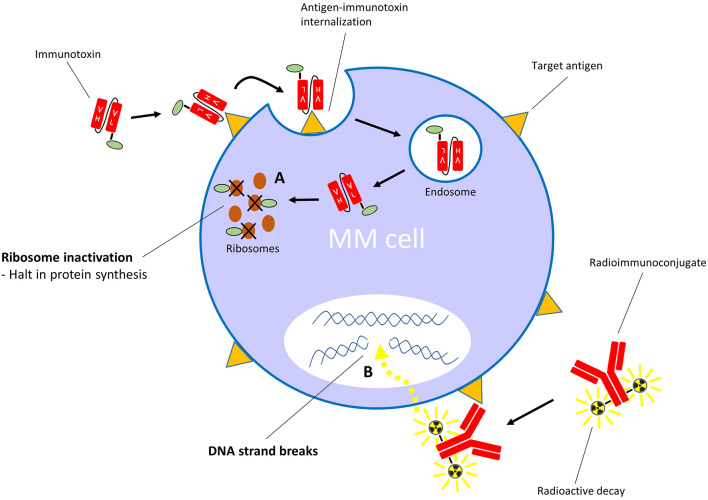
Mechanism of action of immunotoxins with ribosome-inactivating toxins, and radioimmunoconjugates. **(A)** After binding its specific cell surface target, the immunotoxin is internalized into the cell. The toxin moiety then inactivates ribosomes, which leads to inhibition of polypeptide chain elongation. **(B)** The radioimmunoconjugate binds its specific target on the cell surface. Irradiation of cells within the path length of the emitting radionuclide results in DNA strand breaks.

Contrary to ADCs, immunotoxins have potent protein toxins as payloads. To assure specific targeting, the toxin's native binding domain is replaced using genetic engineering techniques by a mAb fragment, or another receptor-specific molecule such as a cytokine or growth factor. The general mechanism of action is similar to ADCs: the targeting moiety binds to a specific target on the cell surface, which is followed by internalization of the immunotoxin, after which the toxin interferes with vital cellular processes (Figure 3). Toxins are highly cytotoxic molecules—internalization of just one molecule into the cytosol is sufficient to induce cell death (98, 99). The majority of toxins used in immunotoxin design are either plant- or bacterial based, such as the bacterial pseudomonas exotoxin, diphtheria toxin and Shiga-like toxin, and the plant toxins ricin, gelonin and pokeweed antiviral protein, among many others (100). The majority of toxins belong to the group of ribosome-inactivating proteins, which induce ribotoxic stress, halt protein production and cause subsequent apoptosis (101). This mechanism of action affects both dividing and non-dividing cells. Additionally, accumulating evidence suggests that immunotoxins can induce anti-tumor immune responses (102). A disadvantage of using toxins as payloads is their natural immunogenicity (103), which causes neutralizing anti-toxin antibody responses when administered to patients. However, toxins can be deimmunized by the removal of critical T cell and B cell epitopes via genetic engineering (104, 105). For an in-depth insight into immunotoxin rationale, design, and advances, we refer to these reviews (100, 106, 107).

The anti-cancer potential of immunotoxins is illustrated by the FDA-approved drug moxetumomab pasudotox (anti-CD22; Lumoxiti) which showed remarkable efficacy as monotherapy in a phase 3 pivotal clinical trial with relapsed/refractory hairy cell leukemia patients, resulting in a 75% ORR including 41.3% complete remission (CR) (108). To date, two additional immunotoxins have been approved by the FDA for the treatment of hematologic malignancies: denileukin difitox (anti-CD25; Ontak; orphan designation granted by EMA) for cutaneous T cell lymphoma and tagraxofusp (anti-CD123; Elzonris; marketing authorization application under review by EMA) for blastic plasmacytoid dendritic cell neoplasm. In the next section, we will focus on the immunotoxins that are or have been under investigation for MM, including one that has recently been translated into a clinical trial.

### Immunotoxins Targeting CD38

#### TAK-169

TAK-169 is a novel type of recombinant immunotoxin, also called an engineered toxin body (ETB) (109, 110). This hybrid molecule is comprised of an anti-CD38 single-chain variable fragment (scFv) fused to the Shiga-like toxin A-subunit (SLTA). CD38 is a transmembrane glycoprotein involved in signaling, adhesion, and ecto-enzymatic processes regulating calcium metabolism (111, 112). CD38 is moderately expressed on many hematopoietic cells including NK cells, monocytes, and a fraction of T cells and B cells. Moreover, it is highly expressed on normal and malignant plasma cells (86, 113, 114), making it an attractive target for anti-MM immunotherapy. Upon binding their target, ETBs force their internalization, and then route retrogradely to the cytosol where they irreversibly inactivate ribosomes resulting in apoptosis. Notably, the SLTA molecules of ETBs are genetically modified to reduce immunogenicity and anti-drug antibody responses, without affecting their potency (115, 116). In preclinical studies, TAK-169 potently and specifically killed CD38-expressing cell lines and primary MM cells in a dose-dependent manner (109). Additional *in vitro* data in MM cell lines showed that short (2 h) exposure to TAK-169 was sufficient for the induction of cytotoxic effects. Notably, TAK-169 binds to a non-overlapping epitope compared with daratumumab, and remains cytotoxic to MM cells *in vitro* in the presence of daratumumab. TAK-169 was also effective against CD38-expressing human MM tumors in mouse models. In these mice, the combination of TAK-169 and ixazomib was more effective when compared with the effects of either drug alone. Furthermore, TAK-169 was well-tolerated in cynomolgus monkeys at doses where pharmacodynamic effects (reduction of CD38-positive NK cells) were detected. TAK-169 monotherapy is now being evaluated in a first-in-human phase 1 dose-escalation and -expansion trial with heavily pretreated relapsed/refractory MM patients (NCT04017130) (110). Patient recruitment has started in late 2019.

#### 1053-PE38

This immunotoxin combines an anti-CD38 single domain antibody (nanobody) with PE38, the 38 kDa version of the pseudomonas exotoxin A (117). 1053-PE38 was highly cytotoxic against MM cell lines in contrast to a non-specific control immunotoxin. Moreover, 1053-PE38 killed primary MM cells isolated from patients in a dose-dependent fashion. Anti-tumor activity was positively correlated with CD38 surface expression levels on target cells. Treatment of target cells with retinoic acid, which increases CD38 expression (118), enhanced their sensitivity to the immunotoxin (117).

#### Other CD38-Targeting Immunotoxins

Table 2 provides an overview of the CD38-targeting immunotoxins OKT10-SAP, HB7-blocked ricin, and IB4/saporin-S6. To the best of our knowledge, except for OKT10-SAP, these agents have not been evaluated in clinical trials.

**Table 2:** List of immunotoxins investigated for multiple myeloma.

**Drug name**	**Target**	**Effector moiety**	**Development state**	**Clinical trial**	**Treatment regimen**	**Status**	**References**
TAK-169	CD38	SLTA (deimmunized)	Clinical	NCT04017130; phase 1	TAK-169 mono	Recruiting	(109, 110)
1053-PE38	CD38	Pseudomonas exotoxin A 38 kDa fragment (PE38)	Preclinical	-	-	-	(117)
IB4/saporin-S6	CD38	Saporin-S6	Preclinical	-	-	-	(119)
OKT10-Sap	CD38	Saporin	Clinical	Cancer Research UK trial; phase 1	OKT10-Sap mono	-	(120)
HB7-blocked ricin	CD38	Ricin	Preclinical	-	-	-	(121)
ch128.1Av/b-SO6 (anti-hTfR IgG3-Av/b-SO6)	CD71	Biotinylated saporin 6	Preclinical	-	-	-	(122)
2L-Rap-hLL1-γ4P	CD74	Frog Rnase	Preclinical	-	-	-	(123)
B-B2-saporinB-B4-saporin	CD138	Saporin	Preclinical	-	-	-	(124)
LMB-70 LMB-75 LMB-38	BCMA (CD269)	Pseudomonas exotoxin A domain III (PE24)	Preclinical	-	-	-	(125–127)
HM1.24-ETA'	CD317	Pseudomonas aeruginosa exotoxin A (ETA)	Preclinical	-	-	-	(128)
rGel/BLyS	BAFF-R (CD268), TACI (CD267) or BCMA (CD269)	Gelonin, recombinant	Preclinical	-	-	-	(129)

*mono, monotherapy.*.

### Immunotoxins Targeting Other Markers

#### Leptomycin B-75 (LMB-75) & Leptomycin B-70 (LMB-70)

These immunotoxins combine the toxin PE24 with either the fragment antigen-binding (Fab) part (LMB-70) or the disulfide-stabilized (ds)Fv part (LMB-75) of the anti-BCMA mAb BM306 (125–127). PE24 consists of domain III of the pseudomonas exotoxin A and has reduced immunogenicity as compared with PE38 (130, 131). LMB-70 dose-dependently decreased the viability of BCMA-positive MM cells while having no effects on BCMA-negative cell lines or non-MM cells from patients. In MM cell lines, similar effects were observed with LMB-75 (126). For LMB-70, 10 min of exposure to the drug was sufficient to kill >95% of MM cells, indicating a rapid, direct toxic effect. Cytotoxicity was associated with cleavage of caspases 3, 8 and 9, and decreased levels of the anti-apoptotic proteins MCL-1 and BCL-XL (125). LMB-70 was also active in MM mouse models (125, 127). Based on the longer half-life of LMB-70 in mice when compared with LMB-75, LMB-70 may be the most promising candidate for further evaluation in clinical trials.

#### HM1.24-ETA'

HM1.24-ETA' is comprised of a CD317-binding scFv linked to a truncated form of the pseudomonas exotoxin A (ETA) (128). CD317 (HM1.24), also called tetherin, is a transmembrane glycoprotein with multiple functions including inhibition of viral particle release from virus-infected cells (132, 133) and activation of the NF-κB pathway (134, 135). CD317 is normally expressed on mature B cells, endothelial cells, monocytes and BMSCs (128, 136–138). Moreover, it is overexpressed on certain cancer cells including MM (139–142). HM1.24-ETA' efficiently and specifically suppressed the growth of MM cells at nanomolar concentrations via the induction of apoptosis (128). The immunotoxin did not affect the viability of CD317-positive BMSCs and monocytes. Decreased monocyte viability was only observed when monocytes were activated by interferon-gamma, which increases their metabolic state. In mice, HM1.24-ETA' abrogated MM tumor growth.

#### Other Immunotoxins

Table 2 summarizes the other immunotoxins that bind CD38, CD138, CD71, CD74, and BAFF receptors.

## Immunocytokines

**Figure 4:**
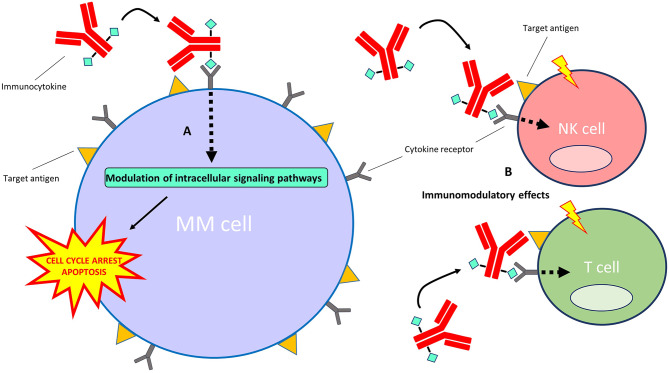
Mechanism of action of immunocytokines. After the immunocytokine binds to its specific target on the cell surface, the cytokine moiety is able to signal via its native cytokine receptor. **(A)** Direct effects on MM cells: the modulation of intracellular signaling pathways by certain cytokines (e.g., interferon-alpha and TRAIL) induces cell cycle arrest and/or apoptosis of MM cells. **(B)** Indirect effects on immune cells: modulation of intracellular signaling pathways by certain cytokines (e.g., interferon-alpha and interleukin-2) results in the stimulation of immune cell subsets, including T cells, NK cells, monocytes/macrophages (not depicted) and dendritic cells (not depicted), in the tumor microenvironment.

Immunocytokines are an emerging treatment modality for auto-immune diseases and cancer (143, 144). They are comprised of mAbs or mAb fragments which are fused to either pro-inflammatory or anti-inflammatory cytokine moieties. Although certain cytokines display substantial direct and indirect anti-cancer effects, their use in the clinic is limited by their short half-life in the circulation and severe dose-dependent adverse effects that occur upon systemic administration of free cytokines in therapeutic relevant doses (145). Immunocytokines intend to increase the therapeutic window compared with free cytokines by specifically increasing cytokine concentrations at tumor sites while limiting off-target toxicities at non-tumor sites. When the mAb or mAb fragment binds to its specific antigen, the conjugated cytokine can signal via its native cytokine-receptor to modulate intracellular signaling pathways (Figure 4). An additional benefit of fusing cytokines to mAbs is their increased half-life in the circulation when compared with free cytokines (146). Also, some immunocytokines retain the ability to induce Fc-mediated effector functions (147). Notably, unmodified cytokines conjugated to mAbs retain a high affinity for their native receptors, which precludes total immunocytokine specificity for the target cells.

Despite their emergence as an immunotherapeutic drug group, to the best of our knowledge, to date no immunocytokine has been approved by the FDA or EMA for the treatment of cancer. However, with regards to MM, several free cytokines such as interferon-alpha (IFNα) and TNF-related apoptosis-inducing ligand (TRAIL) have been extensively studied as a treatment modality because of their known anti-MM effects in preclinical models (148–151). Especially IFNα demonstrates pleiotropic anti-tumor activity by directly inducing apoptosis and growth inhibition of MM cells (152), by indirectly stimulating innate and adaptive immune responses (153, 154) and inhibiting angiogenesis (155, 156). Despite the clinical benefit of adding IFNα to induction treatment or as maintenance therapy in MM patients (157), substantial dose-related side effects resulted in treatment discontinuation in a high proportion of patients (158, 159). However, to reduce toxicity, IFNα can also be conjugated to MM cell-targeting antibodies (147, 160–163).

In the next section, we will discuss five immunocytokines which are or have been under investigation in MM. Table 3 lists three additional anti-MM immunocytokines that bind HLA-DR, CD20/HLA-DR, and fibronectin extra-domain B.

**Table 3:** List of immunocytokines investigated for multiple myeloma.

**Drug name**	**Target**	**Effector moiety**	**Development state**	**Clinical trial**	**Treatment regimen**	**Status**	**References**
TAK-573	CD38	Attenuated IFNα2b (Attenukine™)	Clinical	NCT03215030; phase 1/2	TAK-573 mono and TAK-573 + dex	Recruiting	(162, 164)
IL2-αCD38-αCD38-scTRAIL	CD38	TRAIL & IL-2	Preclinical	-	-	-	(165)
Anti-CD138-IFNα14 Anti-CD138-IFNα2 Anti-CD138-IFNα2^YNS^	CD138	IFNα14 IFNα2 IFNα2^YNS^	Preclinical	-	-	-	(161, 163)
20-C2-2b	CD20 & HLA-DR	IFNα2b	Preclinical	-	-	-	(160)
C2-2b-2b	HLA-DR	Tetrameric IFNα2b	Preclinical	-	-	-	(147)
L19-TNFα L19-IL2	Fibronectin extra-domain B	TFNα IL-2	Preclinical	-	-	-	(166)

*dex, dexamethasone; mono, monotherapy*.

### Immunocytokines Targeting CD38

#### TAK-573

TAK-573 (CD38-Attenukine™) is comprised of a CD38-specific IgG4 mAb fused to an attenuated form of human IFNα2b (162). This mutated IFNα2b component has decreased affinity for the universally expressed IFNα receptor (IFNAR). Accordingly, TAK-573 displays a high specificity for CD38-expressing cells, while having limited activity on CD38-negative cells. *In vitro*, TAK-573 decreased the growth of CD38-positive MM cell lines, while having minimal effects on the viability of CD38-negative cells or the colony formation of normal bone marrow cells. In mouse models, the immunocytokine eliminated CD38-expressing MM and lymphoma tumors, and had synergistic activity when combined with bortezomib or lenalidomide. TAK-573 treated mice showed an influx of antitumor M1 macrophages in the tumor microenvironment. These macrophages were possibly attracted by IFNα-induced chemokines (164). TAK-573 is currently being studied as monotherapy or in combination with dexamethasone in a phase 1/2 trial with heavily pretreated MM patients (NCT03215030).

#### IL2-αCD38-αCD38-scTRAIL

This immunocytokine consists of the anti-CD38 scFv of the mAb daratumumab, in tandem diabody format, concurrently tethered to both interleukin-2 (IL-2) and TRAIL by peptide linkers (165). TRAIL has profound anti-MM activity following interaction with death receptor-4 and -5 (149–151), whereas IL-2 can contribute to antitumor immunity by stimulating cytotoxic CD8-positive T cells and NK cells in the tumor microenvironment (167, 168). IL2-αCD38-αCD38-scTRAIL specifically binds to CD38 on target cells resulting in reduced viability of MM cells (165).

### Other Immunocytokines

#### Anti-CD138-IFNα2, Anti-CD138-IFNα2^YNS^, and Anti-CD138-IFNα14

These immunocytokines combine the specificity of an anti-CD138 IgG1 mAb with the anti-MM activity of an IFNα14, IFNα2, or IFNα2^YNS^ (high-affinity IFNα2 mutant) moiety (161, 163). Preclinical studies showed that these immunocytokines had higher anti-MM activity against MM cell lines compared with an anti-CD20-IFNα2 control immunocytokine. Treatment was associated with a decreased expression of IFN regulatory factor 4 (IRF4), which is a survival factor for MM cells (169, 170). Notably, synergistic activity was observed when immunocytokines were combined with bortezomib (163). In mice, treatment with either immunocytokine reduced MM tumor growth and prolonged survival, and cotreatment of anti-CD138-IFNα14 with bortezomib further improved antitumor activity compared with either treatment alone. Although anti-CD138-IFNα2b ^YNS^ had stronger anti-MM activity at lower concentrations *in vitro* compared with anti-CD138-IFNα2, their efficacy was similar in mice.

## Radioimmunoconjugates

Radioimmunoconjugates, also known as antibody-radionuclide conjugates, consist of a mAb which is linked to a radionuclide (171, 172). Next to their application in cancer imaging (173–175), these radioactive fusion proteins are increasingly explored as a treatment modality for cancer. In contrast to ADCs and immunotoxins, radioimmunoconjugates do not require endocytosis to induce anti-cancer activity. When the mAb fragment binds its specific antigen, the emitting radionuclide causes DNA strand breaks in the target cell (Figure 3). In addition, depending on the path length of the decaying radionuclide, radioimmunoconjugates can also cause radiation of bystander cells via crossfire effects. A variation on this principle is pretargeted radioimmunotherapy (PRIT), in which the (bispecific) antibodies (containing a radionuclide binding site) and radionuclides are separately delivered to the tumor sites in a multi-step approach (176, 177). Anti-MM PRIT agents will not be discussed in detail in this review. As effector moieties, most radioimmunoconjugates contain either alpha particle- or beta particle-emitting radionuclides. Radioimmunoconjugates fused to alpha-emitters are increasingly evaluated for the treatment of hematologic malignancies because of their shorter path length and higher rate of linear energy transfer when compared with beta-emitters. Common toxicities by radioimmunoconjugates are mainly seen in the bone marrow, kidney, and liver. Currently, yttrium-90-ibritumomab tiuxetan (anti-CD20; ^90^Y-Zevalin) is the only radioimmunoconjugate approved by the FDA and EMA for the treatment of follicular B cell NHL. Similar to lymphoma cells, MM cells display inherent sensitivity to radiation (178, 179). In the next section, we will discuss the radioimmunoconjugates which have been assessed in clinical trials, or have shown promise in preclinical models. We also provide an overview of other anti-MM radioimmunoconjugates in Table 4.

**Table 4:** List of radioimmunoconjugates investigated for multiple myeloma.

**Drug name**	**Target**	**Effector moiety**	**Development state**	**Clinical trial**	**Treatment regimen**	**Status**	**References**
Bexxar (iodine-131-tositumomab)	CD20	Iodine-131	Clinical	NCT00135200; phase 2	Bexxar mono as consolidation treatment	Active, not recruiting	(180, 181)
^90^Y-Zevalin (yttrium-90- ibritumomab tiuxetan)	CD20	Yttrium-90	Clinical	NCT00477815; phase 1 NCT01207765; phase 2	^90^Y-Zevalin as part of standard MA ASCT-conditioning ^90^Y-Zevalin as part of standard MA ASCT-conditioning	Completed Terminated (changes in practice)	(182, 183)
Actinium-225-lintuzumab	CD33	Actinium-225	Clinical	NCT02998047; phase 1	Actinium-225-lintuzumab mono	Recruiting	(184)
Actinium-225- daratumumab	CD38	Actinium-225	Preclinical	-	-	-	(185)
Astatine-221- CD38	CD38	Astatine-211	Preclinical	-	-	-	(186)
Bismuth-213- anti-CD38	CD38	Bismuth-213	Preclinical	-	-	-	(187)
Lead-212-daratumumab	CD38	Lead (Pb)-212	Preclinical	-	-	-	(188)
Iodine-131-B-B4	CD138	Iodine-131	Clinical	NCT01296204; phase 1	Iodine-131-B-B4 mono	Completed	(189)
Bismuth-213- B-B4	CD138	Bismuth-213	Preclinical	-	-	-	(190, 191)
Bismuth-213- 9E7.4	CD138	Bismuth-213	Preclinical	-	-	-	(192, 193)
Lutetium-177- 9E7.4	CD138	Lutetium-177	Preclinical	-	-	-	(193)
Bismuth-213- MA5	MUC1	Bismuth-213	Preclinical	-	-	-	(191)

*ASCT, autologous stem cell transplantation; MA, myeloablative; mono, monotherapy*.

### CD38-Targeting Radioimmunoconjugates

#### Actinium-225-Daratumumab

This radioimmunoconjugate is comprised of the anti-CD38 mAb daratumumab conjugated to actinium-225 (185). Actinium-225 is an alpha-emitting radionuclide with a half-life of 10 days which causes double-strand DNA breaks (194). Actinium-225-daratumumab binds to CD38-positive cells with similar affinity as unconjugated daratumumab, and specifically eliminates CD38-expressing MM cell lines *in vitro*. Additionally, the daratumumab-based radioimmunoconjugate retains its full ability to induce ADCC and complement-dependent cytotoxicity. In a MM mouse model, actinium-225-daratumumab had tumor-stabilizing effects at concentrations at which unconjugated daratumumab lacked any activity. Similar to unconjugated daratumumab, the radioimmunoconjugate caused no profound toxicity *in vivo* (185).

#### Astatine-211-OKT10-B10 (^211^At-CD38)

This radioimmunoconjugate is composed of the anti-CD38 IgG1 mAb OKT10 fused to the alpha-emitting radionuclide astatine-221 (186). Similar to actinium-225, astatine-211 causes double-strand DNA breaks but has a considerably shorter half-life of 7.2 h (195). In preclinical evaluation, astatine-211-OKT10-B10 was specifically cytotoxic against CD38-positive MM cells. In mouse models, the radioimmunoconjugate showed beneficial tumor-to-normal-organ ratios of radiation uptake and no signs of significant toxicity. Moreover, astatine-211-OKT10-B10 demonstrated robust and durable disease eradication in disseminated MM models, and delayed tumor growth in subcutaneous MM models (186). O'Steen and coworkers describe a planned phase 1 clinical trial with MM patients in which astatine-211-OKT10-B10 will be evaluated in combination with an ASCT-conditioning regimen.

### Other Radioimmunoconjugates

#### Actinium-225-Lintuzumab

Actinium-225-lintuzumab consists of the humanized CD33-binding mAb lintuzumab coupled to the radionuclide actinium-225 (184). Although the transmembrane receptor CD33 is generally considered a myeloid lineage marker, it is also expressed on malignant plasma cells of 18–33% of MM patients. Moreover, CD33-positive MM is associated with unfavorable cytogenetics and poor prognosis (196–198). Clinical evaluation of actinium-225-lintuzumab monotherapy in a single-center, phase 1 dose-escalation trial with relapsed/refractory MM patients is currently ongoing (184). Patients with ≥25% of MM cells expressing CD33 and with at least three prior lines of treatment are eligible for inclusion.

#### Bexxar (Iodine-131-Tositumomab)

Bexxar comprises the murine anti-CD20 IgG2 mAb tositumomab conjugated to iodine-131 (180, 181). Iodine-131 is a beta-emitting radionuclide commonly used for the treatment of thyroid cancer (199). It has a path length of 1–2 mm and a decay half-life 8 days. CD20 is a cell surface phosphoprotein generally considered as a B cell marker but is also present on malignant plasma cells in ~20% of MM patients (200–202). Single-dose Bexxar monotherapy was evaluated in a single-center phase 2 study as a consolidation approach in MM patients who had achieved at least PR (but no CR) following at least one prior therapy, regardless of CD20 status (181). Preliminary results report the inclusion of 16 patients (median of two lines of prior treatment) of which six patients (38%) had CD20-positive MM. Most reported grade 3–4 adverse events were neutropenia (44%), thrombocytopenia (25%) and infection (12.5%). Bexxar improved response (≥25% reduction in M-protein) in 67% and 10% of patients with CD20-positive or CD20-negative MM, respectively. Two patients with CD20-positive MM achieved a stringent CR. In August 2013, GlaxoSmithKline announced to discontinue the manufacturing of Bexxar, which was followed by a withdrawal of the FDA approval in October 2013 (203).

#### ^90^Y-Zevalin (yttrium-90-ibritumomab tiuxetan)

^90^Y-Zevalin is a fusion protein of the CD20-specific IgG1 mAb ibritumomab, linked to the radionuclide yttrium-90 (182, 183). Yttrium-90 is a beta-emitter with a mean path length of 5 mm and a half-life of 64 hours (204). Thus, ^90^Y-Zevalin kills both CD20-positive MM cells and CD20-negative bystander MM cells. ^90^Y-Zevalin (administered on day−14) was evaluated in a single-center, dose-escalation phase 1 trial in combination with high-dose melphalan (day−2 and−1) prior to ASCT (day 0) in 30 MM patients (183). Three patients experienced a DLT (one at 16 Gy, two at 20 Gy), and all patients experienced grade 4 hematologic adverse events. The MTD of this conditioning regimen was defined as 18 Gy. The ORR was 73%, with 63% achieving a VGPR or better. A subsequent phase 2 trial in which ^90^Y-Zevalin was assessed in MM patients with an incomplete response prior to ASCT was prematurely terminated because of a high rate of bacterial infections (182).

#### Iodine-131-B-B4

In a proof of concept phase 1/2 trial, a single therapeutic dose of the murine anti-CD138 IgG1 mAb B-B4 coupled to iodine-131 was administrated as monotherapy to three relapsed/refractory MM patients who received at least three prior lines of treatment (189). All three patients experienced transient grade 3 or higher hematologic toxicities, but kidney, liver, or thyroid were not significantly affected. No objective responses were observed, although one patient achieved SD.

## Conclusions and Future Perspectives

The outcome of MM patients refractory to IMiDs, PIs, and naked mAbs (daratumumab and elotuzumab) remains poor (7), which underscores the still unmet need for new anti-MM therapies with novel mechanisms of action. A promising approach toward enhancing the power of mAb-based immunotherapy is to arm these antibodies with potent effector moieties. In the field of hemato-oncology, such immunoconjugates are probably the most advanced “magic bullets,” a term coined by Paul Ehrlich in the early 1900s, created to date.

The potential of immunoconjugates is exemplified by the ADC belantamab mafodotin, which induces deep responses with an acceptable toxicity profile as monotherapy in heavily pretreated MM patients, including patients refractory to IMiDs, PIs, and naked anti-CD38 mAbs. Based on these observations, we expect that immunoconjugates will be increasingly evaluated in earlier lines of MM treatment, and that some will become powerful permanent players in the “immunotherapeutic arena.” Importantly, several preclinical studies show that many immunoconjugates have additive or even synergistic activity when combined with IMiDs and PIs. In this respect, belantamab mafodotin is currently being assessed in combination with IMiDs, PIs and novel anti-cancer agents (see Table 1). Notably, many immunoconjugates have non-overlapping toxicity profiles with IMiDs and PIs, which may allow for tolerable combination treatments. Furthermore, it would be of interest to elucidate whether drugs from different immunoconjugate subgroups can be effectively combined. Because some immunotoxins show *in vitro* synergy with microtubule-inhibiting taxanes (125, 205), a similar effect can be envisioned when immunotoxins are combined with ADCs that contain a microtubule-disrupting payload.

Despite their potential as effective anti-MM agents, immunoconjugates also have their limitations. First, immunoconjugates can cause substantial on- or off-target side effects, which is illustrated by eye toxicity induced by belantamab mafodotin and peripheral neuropathy associated with lorvotuzumab mertansine treatment. Notably, not only the type of target, antibody, and linker, but also the type of effector moiety has an impact on the tolerability profile of immunoconjugates. It is therefore important to combine immunoconjugates with anti-MM drugs with non-overlapping toxicity profiles to avoid additive toxicities. We expect that the identification of novel targets and payloads with reduced non-specific uptake may contribute to decreased toxicity of immunoconjugates in the future. Eventually, the comparison of immunoconjugates with different components in preclinical and clinical studies will point out which mechanism of action is most important in eliminating MM cells, while minimizing the effects on normal tissues.

Second, it is important to take into account the rapid clinical development of other forms of immunotherapy, such as the T cell redirecting therapies. Indeed, BCMA-directed CAR-T cells and bispecific antibodies have superior activity as monotherapy in heavily pretreated MM patients, when compared with belantamab mafodotin (64–66, 206). However, in a subset of patients these therapies are associated with substantial side effects, such as cytokine release syndrome and neurotoxicity. Furthermore, CAR-T cells are currently not available “off-the-shelf,” as opposed to mAb-based immunotherapies. Also, in patients with age-, tumor- or therapy-related impairment of the T cell compartment (207–212), CAR-T cells and bispecific antibodies may be less effective than therapies inducing direct cytotoxic effects. It would be of interest to explore whether these ‘immunologically frail' patients benefit more from mAbs armed with powerful effector moieties, such as small molecule drugs, toxins, or radionuclides.

Finally, several mechanisms of resistance may limit the efficacy of immunoconjugates. Target antigen expression is an important factor for target recognition and effector function by mAbs (213). There is considerable heterogeneity in expression of target antigens between MM patients (16, 213–218). Patients with low levels of target antigen on the tumor cell surface are at increased risk of treatment failure (213). Furthermore, MM cells may lack expression of the target antigen [e.g., ~20% of MM is CD56-negative (38, 87, 88)], which will probably be associated with primary resistance to immunoconjugates. Screening for target antigen expression prior to initiation of treatment may be critical in case response is associated with expression levels, and if a substantial fraction of patients lack expression of the target antigen. In addition, progression after prior response may be related to downregulation of the target antigen, which has been shown to contribute to the development of resistance to CD38 antibody treatment (213) and BCMA-targeted T cell redirecting therapy (216, 219, 220). Also, MM is characterized by substantial intraclonal heterogeneity, and outgrowth of a subclone with low target expression may also result in development of disease progression. It is currently unknown to what extent antigen expression levels at baseline and at the time of progression contribute to immunoconjugate-resistance, and further studies are needed to increase our understanding of the impact of antigen expression on primary or acquired resistance to immunoconjugates. Recent studies, especially with ADCs and immunotoxins in other cancers than MM, demonstrate that other potential mechanisms of resistance to immunoconjugates include: (1) increased payload efflux from the cells by drug efflux pumps, such as multidrug resistance protein 1 (MDR1, P-glycoprotein), (2) defects in intracellular trafficking pathways, (3) increased ADC/antigen complex recycling, and (4) activation of anti-apoptotic—or other intracellular signaling pathways (221, 222). Importantly, better insight into those resistance mechanisms may facilitate improvements in immunoconjugate design, and provide a rationale for more efficacious combination strategies. Besides the possible benefit of combining immunoconjugates with IMiDs and PIs, other agents that directly counteract resistance mechanisms may be used. For example, some research groups are exploring the combination of ADCs with MDR1-inhibitors to prevent payload efflux (223–225). As another strategy, immunoconjugates can be combined with drugs that increase the surface expression of the target antigen. For instance, the combination of belantamab mafodotin with a gamma-secretase inhibitor, which increases BCMA density on MM cells, may impede the *in vivo* escape of subclones with low BCMA expression, thereby preventing or delaying disease progression (20). A similar strategy can be envisioned by combining CD38-specific immunoconjugates with agents that increase CD38 expression such as ATRA, panobinostat, or DNA methyltransferases (118, 226, 227). Identification of novel targets for immunoconjugates may also be important to address inter-patient heterogeneity and antigen escape. Potential new targets, such as GPRC5D (215, 217), should be uniformly expressed on MM cells, and not on critical healthy tissues.

In summary, immunoconjugates are versatile immunotherapeutic agents with pleiotropic mechanisms of action. Depending on the effector moiety and mAb characteristics, these may include direct pro-apoptotic/anti-proliferative effects, indirect Fc-mediated effects (ADCC and ADCP), and indirect immunomodulatory effects. The ADC belantamab mafodotin is the first in its class to show potent anti-MM activity in clinical trials (17–19). Encouraging results from the ever-growing list of preclinical and clinical studies with immunoconjugates demonstrate that this drug group holds promise as a treatment strategy for MM patients. Moreover, the immunoconjugate development field is rapidly progressing with the identification of novel targets, and improvements of payloads and linkers. We expect that targeted therapy with immunoconjugates will be increasingly used in clinical trials, and, soon upon regulatory approval, also outside clinical studies.

## Author Contributions

WB (literature research) and ND (supervision) prepared the first draft of the manuscript. TM and SZ critically reviewed the manuscript. All authors edited the manuscript and checked the final version of it.

## Conflict of Interest

SZ has received research support from Celgene, Janssen Pharmaceuticals and Takeda; and serves in advisory boards for Celgene, Janssen Pharmaceuticals, Takeda, Amgen, and Sanofi. TM has received research support from Janssen Pharmaceuticals, Genmab, Takeda, Onkimmune, and Gadeta. ND has received research support from Janssen Pharmaceuticals, AMGEN, Celgene, Novartis, and BMS; and serves in advisory boards for Janssen Pharmaceuticals, AMGEN, Celgene, BMS, Takeda, Roche, Novartis, Bayer, and Servier. The remaining authors declare that the research was conducted in the absence of any commercial or financial relationships that could be construed as a potential conflict of interest.
